# Lack of Association of SULT1A1 R213H Polymorphism with Colorectal Cancer: A Meta-Analysis

**DOI:** 10.1371/journal.pone.0019127

**Published:** 2011-06-13

**Authors:** Chun Zhang, Jian-Ping Li, Guo-Qiang Lv, Xian-Min Yu, Yuan-Long Gu, Ping Zhou

**Affiliations:** 1 Department of General Surgery, The Third Affiliated Hospital to Nantong University, Wuxi, China; 2 Intensive Care Unit, The Third Affiliated Hospital to Nantong University, Wuxi, China; Ulm University, Germany

## Abstract

**Background:**

A number of case-control studies were conducted to investigate the association of SULT1A1 R213H polymorphisms with colorectal cancer (CRC) in humans. But the results were not always consistent. We performed a meta-analysis to examine the association between the SULT1A1 R213H polymorphism and CRC.

**Methods and Findings:**

Data were collected from the following electronic databases: PubMed, Elsevier Science Direct, Excerpta Medica Database, and Chinese Biomedical Literature Database, with the last report up to September 2010. A total of 12 studies including 3,549 cases and 5,610 controls based on the search criteria were involved in this meta-analysis. Overall, no significant association of this polymorphism with CRC was found (H versus R: OR = 1.04, 95%CI = 0.94–1.16, *P* = 0.46; HR+HH versus RR: OR = 1.01, 95%CI = 0.92–1.11, *P* = 0.81; HH versus RR+HR: OR = 1.01, 95%CI = 0.74–1.38, *P* = 0.95; HH versus RR: OR = 1.00, 95%CI = 0.77–1.31, *P* = 0.98; HR versus RR: OR = 1.01, 95%CI = 0.92–1.11, *P* = 0.86). In subgroup analysis, we also did not find any significant association in Cauasians (H versus R: OR = 1.02, 95%CI = 0.92–1.15, *P* = 0.68; HR+HH versus RR: OR = 0.99, 95%CI = 0.91–1.09, *P* = 0.90; HH versus RR+HR: OR = 1.01, 95%CI = 0.73–1.39, *P* = 0.97; HH versus RR: OR = 0.99, 95%CI = 0.75–1.31, *P* = 0.94; HR versus RR: OR = 0.99, 95%CI = 0.90–1.09, *P* = 0.85). The results were not materially altered after the studies which did not fulfill Hardy-Weinberg equilibrium were excluded (H versus R: OR = 1.06, 95%CI = 0.95–1.19, *P* = 0.31; HR+HH versus RR: OR = 1.03, 95%CI = 0.93–1.13, *P* = 0.56; HH versus RR+HR: OR = 1.10, 95%CI = 0.78–1.56, *P* = 0.57; HH versus RR: OR = 1.09, 95%CI = 0.83–1.44, *P* = 0.53; HR versus RR: OR = 1.02, 95%CI = 0.92–1.13, *P* = 0.75).

**Conclusion:**

This meta-analysis demonstrates that there is no association between the SULT1A1 R213H polymorphism and CRC.

## Introduction

Colorectal cancer (CRC) is cancer of the colon or rectum, and it is equally common in men and women. With 655,000 deaths worldwide per year, it is the third leading cause of cancer-related death in the world [1], [2]. In 2010, there were 142,570 new CRC cases and 51,370 deaths from CRC in the United States [2]. Currently, CRC is a major public health burden in many countries. Thus an understanding of the causes of this disease is an area of intense interest. Current evidence supports an important role for genetics in determining risk for CRC [3].

Sulfotransferases (SULTs) play an important role in normal physiological process and malignant transformation [4]. In humans, there are three members of the phenol sulfotransferase family (SULT1A1, SULT1A2, and SULT1A3). SULT1A1 is expressed in the liver as well as in many extrahepatic tissues including colonic mucosa, and is an component in the detoxification pathway of numerous xenobiotics [5], [6]. It plays an important role in the metabolism and bioactivation of many dietary and environmental mutagens, including heterocyclic amines implicated in carcinogenesis of colorectal and other cancers [7], [8]. Hence, SULT1A1 gene may be a good candidate for genetics studies on CRC.

The SULT1A1 gene is located on chromosome 16p12.1-p11.2 [9]. A polymorphism (R213H) in the SULT1A1 gene has been identified in the coding region at nucleotide 638 (a G to A transition). This base change results in a change in the amino acid sequence from arginine to histidine (Arg213His), leading to a decrease in enzymatic activity [10].

Since its discovery in 1997, this polymorphism has attracted widespread attention, and a number of case-control studies were conducted to investigate the association of this polymorphism with CRC in humans [11]–[24]. But the results are not always consistent. There are several possible explanations for this discordance, such as small sample size, ethnic background, uncorrected multiple hypothesis testing, and publication bias. Meta-analysis is a statistical procedure for combining the results of several studies to produce a single estimate of the major effect with enhanced precision [25]. It has become important in cancer genetics because of rapid increases in the number and size of datasets. The aim of the present study is to perform a comprehensive meta-analysis to evaluate the association between the SULT1A1 R213H polymorphism and CRC.

## Methods

### Search strategy

In this meta-analysis, we performed an exhaustive search on studies that examined the association of the SULT1A1 gene polymorphisms with CRC. Data were collected from the following electronic databases: Pubmed, Elsevier Science Direct, Excerpta Medica Database (Embase), and Chinese Biomedical Literature Database (CBM). We searched the articles using the search terms “sulfotransferase”, “SULT”, “SULT1A1”, “colorectal”, “colon”, “rectum”, and “colorectum”. Additional studies were identified by a hand search of references of original studies and review articles on the association between the SULT1A1 R213H polymorphism and CRC. No language restrictions were applied. A study was included in the current meta-analysis if (1) it was published up to September, 2010; (2) it was a case-control study of the SULT1A1 R213H polymorphism and CRC. We excluded the study in which family members were studied. When there were multiple studies from the same population, only the largest study was included.

Furthermore, two investigators independently searched the electronic databases. An independent PubMed search was done (by Zhou P and Zhang C) with the same method. An independent Elsevier Science Direct search was done (by Lv GQ and Yu XM) with the same method. An independent Embase search was done (by Gu YL and Li JP) with the same method. An independent CBM search was done (by Lv GQ and Yu XM) with the same method. References in original studies and review articles were reviewed (by Gu YL and Li JP) to identify additional studies.

### Data extraction

Two investigators (Zhou P and Zhang C) independently extracted data and reached consensus on the following characteristics of the selected studies: the first author's name, year of publication, source of publication, ethnicity, number of cases and controls, and available allele and genotype frequencies information. If original data was unavailable in articles, a request for original data was sent to the corresponding author.

### Statistical analysis

The strength of association between the SULT1A1 R213H polymorphism and CRC was accessed by calculating odds ratio (OR) with 95% confidence interval (CI). We evaluated the allele contrast (H versus R), the codominant model (HH versus RR, HR versus RR), the dominant model (HR+HH versus RR) and the recessive model (HH versus RR+HR), respectively. The heterogeneity between the studies was assessed by the Chi square-test based Q-statistic [26]. A significant Q-statistic (*P*<0.10) indicated heterogeneity across studies. We also measured the effect of heterogeneity by another measure, *I^2^* = 100%×(Q-df)/Q [27]. The pooled OR was calculated by a fixed effect model (using the Mantel-Haenszel method) or a random effect model (using the DerSimonian-Laird method) according to the heterogeneity among studies [28], [29]. The potential publication bias was estimated using Egger's linear regression test by visual inspection of the Funnel plot [30]. In addition, a Chi square-test was used to determine if observed frequency of genotype in control population conformed to Hardy-Weinberg equilibrium (HWE) expectations. Analyses were performed using the software Review Manager 4.2 (Cochrane Collaboration, http://www.cc-ims.net/RevMan/relnotes.htm/) and Stata version 10 (StataCorp LP, College Station, Texas, USA). A *P* value less than 0.05 was considered statistically significant, and all the *P* values were two sided.

## Results

### Characteristics of eligible studies

**Figure 1:**
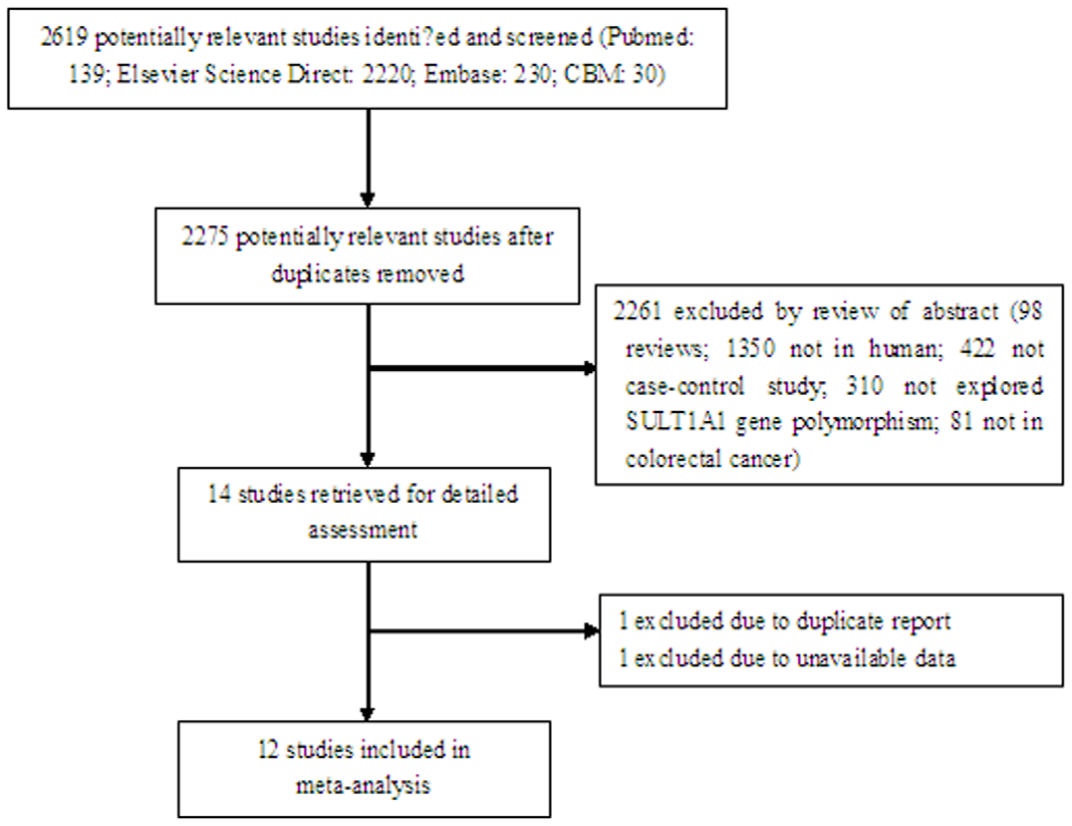
Flow diagram of the study selection process.

Characteristics of studies included in the current meta-analysis are presented in Table 1 [11]–[22]. There were 2619 papers relevant to the searching terms (Pubmed: 139; Elsevier Science Direct: 2220; Embase: 230; CBM: 30). The study selection process is shown in Figure 1. A total of 14 studies examined the association between the SULT1A1 R213H polymorphism and CRC [11]–[24]. Of these, 2 were excluded (the data of 1 study was unavailable, 1 was duplicate report) [23], [24]. Thus, 12 studies were included in the current meta-analysis [11]–[22].

**Table 1:** Characteristics of studies included in the meta-analysis.*

ID	Study	Year	Ethnic group	Sample size (Frequency of H allele, %)		OR (95%CI) for H versus R allele	Hardy-Weinberg equilibrium of genotype of control
				Case	Control		
1	Cotterchio et al. [11]	2008	mainly Caucasian	834(31.35)	1249(32.78)	0.936(0.820–1.070)	0.078
2	Lilla et al. [12]	2007	Caucasian	504(35.61)	603(34.91)	1.031(0.866–1.229)	0.178
3	Chen et al. [13]	2006	Asian	140(8.93)	343(6.27)	1.466(0.877–2.451)	0.749
4	Gaustadnes et al. [14]	2006	Caucasian	230(37.39)	540(32.69)	1.230(0.980–1.544)	NA
5	Sun et al. [15]	2005	Caucasian	109(48.17)	666(37.31)	1.561(1.171–2.082)	0.478
6	Landi et al. [16]	2005	Caucasian	361(26.59)	320(26.88)	0.986(0.775–1.254)	0.011
7	Sterjev et al. [17]	2005	Caucasian	100(27.00)	200(36.75)	0.637(0.439–0.924)	0.034
8	Pereira et al. [18]	2005	Brazilian	42(36.90)	100(33.00)	1.188(0.697–2.022)	0.960
9	Sachse et al. [19]	2002	Caucasian	490(34.39)	593(32.12)	1.107(0.925–1.325)	0.734
10	Nowell et al. [20]	2002	Caucasian	130(37.31)	301(42.36)	0.801(0.601–1.092)	0.815
11	Wong et al. [21]	2002	Caucasian	383(30.94)	402(32.09)	0.948(0.766–1.173)	0.082
12	Bamber et al. [22]	2001	Caucasian	226(34.51)	293(32.08)	1.116(0.860–1.447)	0.621

*OR: odds ratio; CI: confidence interval; NA: not available.

12 studies consisted of 10 Caucasian, 1 Asian and 1 Brazilian. The allele and the genotype frequencies of the SULT1A1 R213H polymorphism were extracted from 11 studies. But only allele frequency was extracted from the study by Gaustadnes et al. [14]. Therefore, examining the contrast of HH versus RR, HR versus RR, HR+HH versus RR, and HH versus RR+HR, the meta-analysis was performed with 11 studies overall, and 9 studies Caucasian. Examining the contrast of H allele versus R allele, meta-analysis was performed with 12 studies overall, and 10 studies Caucasian.

The results of HWE test for the distribution of the genotype in control population are shown in Table 1. Two studies were not in HWE in eligible studies [16], [17]. It was unavailable for one study to perform HWE test [14].

### Meta-analysis

The main results of this meta-analysis and the heterogeneity test are shown in Table 2.

**Table 2:** Meta-analysis of the association of SULT1A1 R213H polymorphism and colorectal cancer.*

Polymorphism	Study	Sample size		No. of Studies	Test of association					Test of heterogeneity	
		Case	Control		*OR (95%CI)*	*Z*	*P-value*	*Model*	*χ^2^*	*P-value*	*I^2^(%)*
H vs R	Overall	7098	11220	12	1.04(0.94–1.16)	0.75	0.46	R	25.00	0.009	56.0
	HWE	5716	9100	9	1.06(0.95–1.19)	1.01	0.31	R	16.13	0.04	50.4
	Caucasian	6734	10334	10	1.02(0.92–1.15)	0.41	0.68	R	22.83	0.007	60.6
HR+HH vs RR	Overall	3319	5070	11	1.01(0.92–1.11)	0.24	0.81	F	8,53	0.58	0.0
	HWE	2858	4550	9	1.03(0.93–1.13)	0.58	0.56	F	5.31	0.72	0.0
	Caucasian	3137	4627	9	0.99(0.91–1.09)	0.12	0.90	F	5.53	0.70	0.0
HH vs RR+HR	Overall	3319	5070	11	1.01(0.74–1.38)	0.07	0.95	R	37.81	<0.0001	73.6
	HWE	2858	4550	9	1.10(0.78–1.56)	0.57	0.57	R	32.46	<0.0001	75.4
	Caucasian	3137	4627	9	1.01(0.73–1.39)	0.04	0.97	R	37.34	<0.0001	78.6
HH vs RR	Overall	1945	2999	11	1.00(0.77–1.31)	0.03	0.98	R	25.00	0.005	60.0
	HWE	1648	2666	9	1.09(0.83–1.44)	0.62	0.53	R	19.24	0.01	58.4
	Caucasian	1809	2641	9	0.99(0.75–1.31)	0.07	0.94	R	24.55	0.002	67.4
HR vs RR	Overall	2948	4482	11	1.01(0.92–1.11)	0.18	0.86	F	10.75	0.38	6.9
	HWE	2528	4028	9	1.02(0.92–1.13)	0.32	0.75	F	9.68	0.29	17.3
	Caucasian	2771	4051	9	0.99(0.90–1.09)	0.19	0.85	F	7.68	0.46	0.0

*SULT1A1: sulfotransferase 1A1; vs: versus; R: random effect model; F: fixed effect model; HWE: Hardy-Weinberg equilibrium.

### Analysis in overall population

The association between the SULT1A1 R213H polymorphism and CRC was investigated in 12 studies with a total of 3,549 cases and 5,610 controls. We detected significant between-study heterogeneity in the contrasts of H versus R, HH versus RR+HR, and HH versus RR. We found no association between the SULT1A1 R213H polymorphism and CRC in overall population (H versus R: OR = 1.04, 95%CI = 0.94–1.16, *P* = 0.46; HR+HH versus RR: OR = 1.01, 95%CI = 0.92–1.11, *P* = 0.81; HH versus RR+HR: OR = 1.01, 95%CI = 0.74–1.38, *P* = 0.95; HH versus RR: OR = 1.00, 95%CI = 0.77–1.31, *P* = 0.98; HR versus RR: OR = 1.01, 95%CI = 0.92–1.11, *P* = 0.86).

### Analysis in HWE population

Meta-analysis was carried out in those studies fulfilling HWE. The meta-analysis included 9 studies (2,858 cases and 4,550 controls). The Q-test of heterogeneity was significant in the contrasts of H versus R, HH versus RR+HR, and HH versus RR. We did not detect an association of the SULT1A1 R213H polymorphism and CRC in HWE population (H versus R: OR = 1.06, 95%CI = 0.95–1.19, *P* = 0.31; HR+HH versus RR: OR = 1.03, 95%CI = 0.93–1.13, *P* = 0.56; HH versus RR+HR: OR = 1.10, 95%CI = 0.78–1.56, *P* = 0.57; HH versus RR: OR = 1.09, 95%CI = 0.83–1.44, *P* = 0.53; HR versus RR: OR = 1.02, 95%CI = 0.92–1.13, *P* = 0.75).

### Analysis in Caucasian population

The meta-analysis included 10 studies (3,367 cases and 5,167 controls) in Caucasian population. The Q-test of heterogeneity was significant in the contrasts of H versus R, HH versus RR+HR, and HH versus RR. No statistically significant association was established for the SULT1A1 R213H polymorphism in Caucasian population (H versus R: OR = 1.02, 95%CI = 0.92–1.15, *P* = 0.68; HR+HH versus RR: OR = 0.99, 95%CI = 0.91–1.09, *P* = 0.90; HH versus RR+HR: OR = 1.01, 95%CI = 0.73–1.39, *P* = 0.97; HH versus RR: OR = 0.99, 95%CI = 0.75–1.31, *P* = 0.94; HR versus RR: OR = 0.99, 95%CI = 0.90–1.09, *P* = 0.85).

### Evaluation of publication bias

The shapes of the funnel plots did not reveal any evidence of obvious asymmetry (funnel plots not shown). Meanwhile, we assessed funnel plot asymmetry by the method of Egger's linear regression test. The intercept *a* provides a measure of asymmetry, and the larger its deviation from zero the more pronounced the asymmetry. The results of Egger's linear regression test are shown in Table 3. It was shown that there was no publication bias for all comparisons.

**Table 3:** Egger's linear regression test to measure the funnel plot asymmetric.*

Comparisons	Y axle intercept: *a (95%CI)*				
	H vs R	HR+HH vs RR	HH vs RR+HR	HH vs RR	HR vs RR
Overall	0.75(−2.03∼3.55)	0.48(−1.29∼2.25)	−0.30(−3.66∼3.04)	−0.13(−2.87∼2.60)	0.04(−1.96∼2.05)
HWE	1.67(−1.07∼4.43)	1.14(−0.34∼2.63)	0.36(−3.60∼4.32)	0.55(−2.45∼3.57)	0.37(−2.02∼2.76)
Caucasian	0.01(−4.32∼4.36)	−0.74(−2.94∼1.45)	−1.17(−7.36∼5.01)	−1.16(−6.13∼3.80)	−1.40(−3.68∼0.87)

*P>0.05; vs: versus; HWE: Hardy-Weinberg equilibrium.

## Discussion

Multiple lines of evidence support an important role for genetics in determining risk for cancer, and association studies are appropriate for searching susceptibility genes involved in cancer [31]. Nevertheless, small sample sized association studies lack statistical power and have resulted in apparently contradicting findings [32]. Meta-analysis is a means of increasing the effective sample size under investigation through the pooling of data from individual association studies, thus enhancing the statistical power of the analysis for the estimation of genetic effects [25]. In the current meta-analysis, on the basis of 12 case-control studies providing data on the SULT1A1 R213H polymorphism and CRC involving 3,549 cases and 5,610 controls, we did not find any significant association between the SULT1A1 R213H polymorphism and CRC among overall and Caucasian populations. Moreover, the results were not materially altered after the studies which did not fulfill HWE were excluded. Our meta-analysis suggests that the SULT1A1 R213H polymorphism is not associated with CRC development. As far as we know, this is the first meta-analysis carried out so far aimed at investigating the association of the SULT1A1 R213H polymorphism with CRC.

SULT1A1 are associated with the detoxification and activation of different carcinogens, and the regulation of many hormones [7], [8]. It has been observed that a G to A transition at nucleotide 638 in SULT1A1 gene causes an Arg to His substitution associated with a low enzymatic activity [10]. Recently, studies have suggested that SULT1A1 HH genotype was associated with an increased risk for some cancers development, such as esophagus, breast, and lung cancer [33]–[35]. These results seem to support that low activity of SULT1A1*H allozyme lacks a protection against dietary and/or environmental chemicals involved in the carcinogenesis of cancer. However, our meta-analysis suggests that the SULT1A1 R213H polymorphism is not associated with CRC risk. The study by Raftogianis et al. [10] suggests that this polymorphism is associated with a low enzyme activity. The enzyme activity was measured using platelet preparations in this study. Nevertheless, the use of platelets to determine the activity of a specific enzyme can be misinterpreted due to the inability of the method to distinguish which enzyme is responsible for the activity observed [21]. Moreover, preliminary modelling studies indicate that this polymorphism does not appear to directly affect the binding site of the substrate or of the universal sulphonate donor 3′phosphoadenosine-5′-phosphosulphate (PAPS) [36]. Meanwhile, in the study performed by Ozawa et al. [37], although a recombinant enzyme was used, differences in sulphonating abilities were only slight, which may be explained by the compromise in thermostability the amino acid change causes. In addition, evdiences suggest that the SULT1A1 R213H polymorphism is in linkage disequilibrium with SULT1A2 N235T [38], [39]. Thus, it is possible that the activity measured in their study is attributable to SULT1A2 and SULT1A1. Further studies of the functional implications of this polymorphism are still needed in the future.

Several specific details merit consideration in the current meta-analysis. Firstly, a recent meta-analysis showed that the SULT1A1 R213H polymorphism had no exact effect to increase the risk of breast cancer, but it did increase the risk of breast cancer among postmenopausal women [40]. Therefore, this polymorphism may only play a role in conjunction with environmental exposures. A more precise analysis stratified by environmental exposures could be performed if individual data were available. Secondly, it is worth mentioning that only 2 of the 12 studies were conducted in non-Cauasian population in our meta-analysis. Hence, the results of our meta-analysis indicate that there is no association between the SULT1A1 R213H polymorphism and CRC, mainly in Cauasian population. Thirdly, significant between-study heterogeneity was detected in some comparisons, and may be distorting the meta-analysis. Fouthly, only published studies were included in this meta-analysis, and publication bias may occur. Fifthly, our results should be interpreted with caution because the prevalence of the SULT1A1 R213H polymorphism may be different in various subtypes of CRC. A analysis stratified by different subtypes of CRC may provide a more precise result. Finally, although we minimized the likelihood of bias by developing a detailed protocol before initiating the study, meta-analysis remains retrospective research that is subject to the methodological deficiencies of the included studies.

In conclusion, our meta-analysis demonstrates that there is no association between the SULT1A1 R213H polymorphism and CRC, mainly in Cauasian population. To reach a definitive conclusion, further gene-gene and gene-environment interactions studies based on larger sample size are still needed, especially in non-Cauasian population.

## Supporting Information

Checklist S1 (DOC)
